# Successful Catheter Ablation of Ventricular Tachycardia Precipitated by Cardiac Resynchronization Therapy

**DOI:** 10.19102/icrm.2023.14038

**Published:** 2023-03-15

**Authors:** Muhammad Atif Rauf, Manish Kalla, Francisco Leyva

**Affiliations:** ^1^Queen Elizabeth Hospital Birmingham, Birmingham, UK

**Keywords:** Cardiac resynchronization therapy, catheter ablation, ventricular tachycardia

## Abstract

A patient with ischemic cardiomyopathy and an implantable cardioverter-defibrillator underwent an upgrade with an epicardial left ventricular lead, which precipitated recurrent ventricular tachycardia (VT). An electrophysiological study with electroanatomic mapping showed the site of the left ventricular lead to be part of the re-entrant circuit, and substrate modification of an endocardial channel led to the resolution of VT and an improvement in symptoms.

## Case presentation

**Figure 1:**
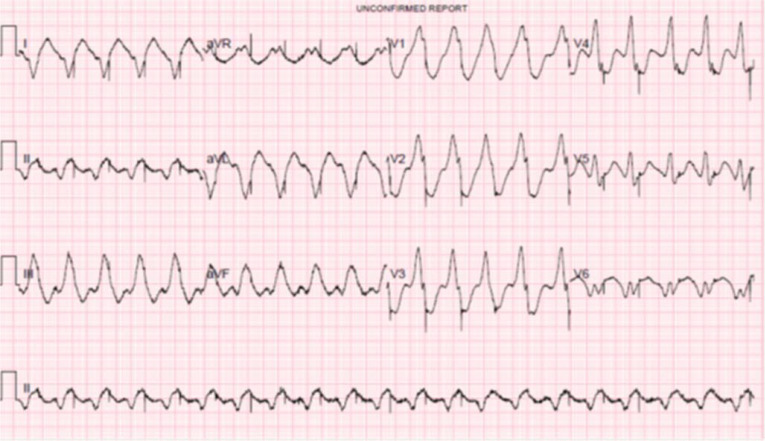
Clinical ventricular tachycardia (precipitated by the left ventricular lead).

A 69-year-old man with ischemic cardiomyopathy with severe left ventricular (LV) dysfunction and an implantable cardioverter-defibrillator (ICD) implanted 8 years prior was evaluated for worsening heart failure (HF). He underwent an ICD to cardiac resynchronization therapy defibrillator (CRT-D) upgrade with an active quadripolar LV lead, which was placed successfully in the posterolateral branch of the coronary sinus. The sensing and pacing parameters in that position were good, and there were no immediate complications. One week later, the patient presented with an electrical storm, which was attributed to the LV lead. Device interrogation showed multiple episodes of monomorphic ventricular tachycardia (VT) **(Figure 1)**. Further episodes of VT with the same morphology persisted despite treatment with amiodarone and switching off/on the LV lead. Switching off the LV lead led to the deterioration of HF symptoms and an increase in QRS duration to 157 ms.

**Figure 2:**
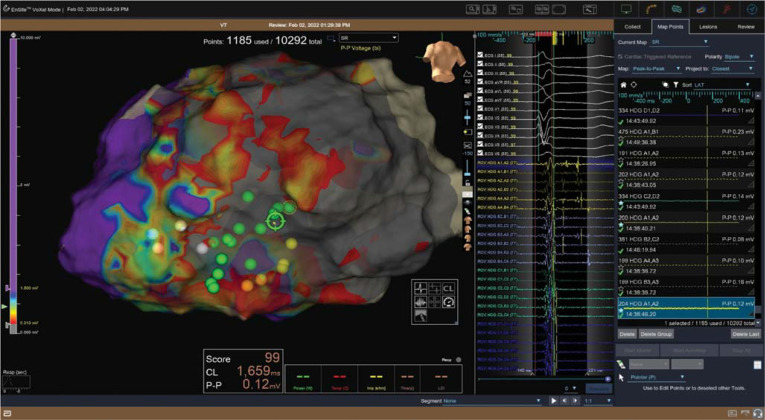
Late potentials.

**Figure 3:**
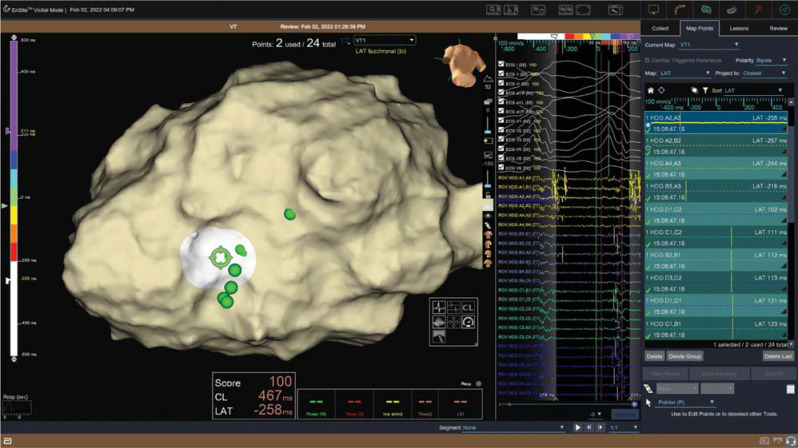
Signals during ventricular tachycardia.

**Figure 4:**
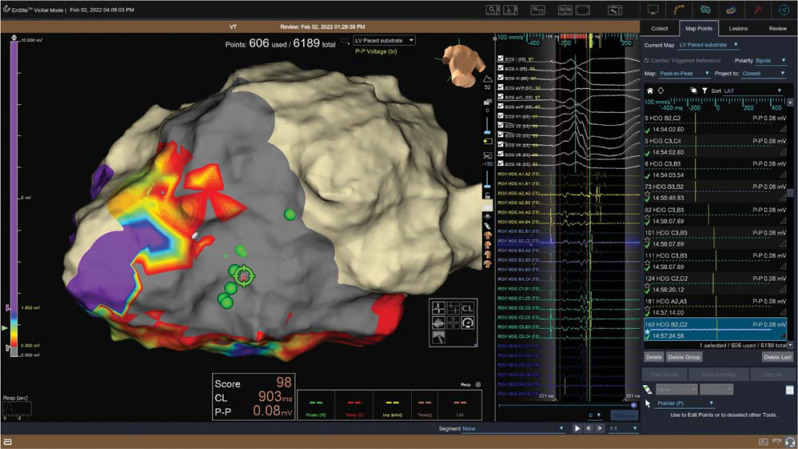
Late potentials during left ventricular pacing.

After discussion with the multidisciplinary team, endocardial VT ablation was undertaken after obtaining high-risk informed written consent from the patient. Following right femoral venous access and anticoagulation, an uncomplicated transseptal puncture was performed and a large curve steerable sheath (Agilis™; Abbott, Chicago, IL, USA) was passed into the left ventricle. We mapped the left ventricle using the Advisor™ HD Grid mapping catheter (Abbott) via the EnSite Precision 3-dimensional mapping system (Abbott). This demonstrated a large inferolateral scar, extending from the base down to the apex, and, interestingly, there was a channel of late potentials crossing this scar **(Figure 2)**. During the spontaneous initiation of the patient’s clinical VT, an early signal possibly representing an entry point was seen in the area of annotated late potentials **(Figure 3)**. We repeated the mapping with LV-only pacing again, identifying late conduction through this area, even though the pacing site was near, with conduction wrapping around this area of the scar **(Figure 4 and Video 1)**. Given the fact that we were unable to induce VT from the right ventricle, left ventricle, or with an LV lead non-invasive programmed stimulation, we proceeded to a substrate modification through this area, effectively transecting the channel and extending the lesion set to anchor it to the border zones of the scar. Following this, no VT was inducible with programmed electrical stimulation. Given the patient’s frailty, this was accepted as a reasonable endpoint. We switched back to his biventricular pacing. There were no post-procedural complications. The patient improved remarkably in the ensuing weeks, and device interrogation showed 98.2% biventricular pacing and no monitored or treated ventricular arrhythmias.

## Discussion

CRT has been proven to reduce mortality in HF patients.^1^ CRT-induced pro-arrhythmia is a rare but life-threatening complication of CRT implantation often resulting in an electrical storm. The electrical storm is usually refractory to anti-arrhythmic drug therapy. Inactivation of biventricular pacing is undertaken as a last resort in these patients, which can lead to the progression of HF and induction of cardiogenic shock. The prognosis becomes poor in these patients, with a mortality rate of about 50%.^2^

The possible mechanisms of CRT-induced pro-arrhythmias include prolongation of repolarization induced by the epicardial LV lead or the facilitation of re-entry circuits by the LV lead.^3^ Roque et al.^4^ studied endocardial and epicardial maps in 64 patients undergoing VT ablation. A re-entry circuit was the underlying mechanism among all patients presenting with CRT-associated pro-arrhythmia. In addition, analyses of the relationship between electroanatomic maps and the fluoroscopic position of the LV lead demonstrated that the epicardial lead was located within the scar in 80% of CRT-associated pro-arrhythmia cases, and this occurrence was significantly more common compared to in patients without CRT-associated pro-arrhythmia. Ablation with substrate modification within the epicardial scar near the LV lead was successfully undertaken, and patients were able to successfully resume biventricular pacing in most cases. This study demonstrated that the spatial relationship of LV lead positioning in or near the epicardial scar is critical for the pro-arrhythmic effects of CRT. Furthermore, the presentation of an electrical storm was found to be an early event following CRT device implantation, which also demonstrates that it is linked to LV pacing rather than the progression of HF or underlying cardiomyopathy.^4^ Our patient’s presentation with electrical storm, which was only 1 week after the CRT upgrade, also highlights the same mechanism.

Animal studies have demonstrated that epicardial activation versus endocardial activation of the LV wall leads to greater prolongation of the QT interval and transmural dispersion of repolarization, leading to pro-arrhythmic effects.^5^ This can lead to the initiation of torsades de pointes and has been reported in humans after implantation of CRT devices.^6^ In our case, the site of LV lead was putatively part of the re-entry circuit of the VT, which was ablated successfully by substrate modification via an endocardial approach.

## Conclusion

We present an unusual case of a patient in which an upgrade to a CRT device with an LV lead precipitated recurrent VT. The site of the LV lead was shown to be part of the re-entrant circuit, and substrate modification in this area led to the resolution of VT and the reinstitution of biventricular pacing.

## Figures and Tables

**Video 1:** Left ventricular pacing propagation.
